# Community Outbreak of *Pseudomonas aeruginosa* Infections Associated with Contaminated Piercing Aftercare Solution, Australia, 2021

**DOI:** 10.3201/eid2910.230560

**Published:** 2023-10

**Authors:** Benjamin T. Trevitt, Anthea L. Katelaris, Catherine Bateman-Steel, Sandra Chaverot, Sinead Flanigan, Toni Cains, Elena Martinez, Andrew Ginn, Vitali Sintchenko, Arthur Jones, Kishen Lachireddy, Mark J. Ferson, Vicky Sheppeard

**Affiliations:** South Eastern Sydney Public Health Unit, Sydney, New South Wales, Australia (B.T. Trevitt, A.L. Katelaris, C. Bateman-Steel, S. Chaverot, S. Flanigan, T. Cains, M.J. Ferson, V. Sheppeard);; University of Sydney, Sydney (B.T. Trevitt, E. Martinez, A. Ginn, V. Sintchenko, V. Sheppeard);; Institute for Clinical Pathology and Medical Research New South Wales Health Pathology, Sydney (E. Martinez, A. Ginn, V. Sintchenko);; St George Hospital, Sydney (A. Jones);; Health Protection NSW, Sydney (K. Lachireddy);; University of New South Wales, Sydney (M.J. Ferson)

**Keywords:** *Pseudomonas aeruginosa*, outbreak, piercing, genomics, infection, communicable diseases, communicable disease control, public health, aftercare, bacteria, Australia

## Abstract

In April 2021, the South Eastern Sydney Local Health District Public Health Unit (Sydney, New South Wales, Australia) was notified of 3 patients with *Pseudomonas aeruginosa* infections secondary to skin piercings performed at the same salon. Active case finding through laboratories, clinician alerts, and monitoring hospital visits for piercing-related infections identified additional cases across New South Wales, and consumers were alerted. We identified 13 confirmed and 40 probable case-patients and linked clinical isolates by genomic sequencing. Ten confirmed case-patients had used the same brand and batch of aftercare solution. We isolated *P. aeruginosa* from opened and unopened bottles of this solution batch that matched the outbreak strain identified by genomic sequencing. Piercing-related infections returned to baseline levels after this solution batch was recalled. Early outbreak detection and source attribution via genomic sequencing are crucial for controlling outbreaks linked to contaminated products. Manufacturing standards for nonsterile cosmetic products and guidance for piercing aftercare warrant review.

Sporadic bacterial infections are a relatively common occurrence after nonmedical body piercing procedures, such as ear piercing (*1*). Localized infections occur at 10%–30% of new piercing sites (*1*), most commonly caused by *Staphylococcus aureus*, *Streptococcus pyogenes*, or *Pseudomonas aeruginosa* (*2*). Complications of piercing-related infections can range from minor superficial skin infections to abscess formation and necrosis requiring surgical intervention (*2*). Severe infections tend to occur in more avascular areas, such as auricular cartilage (*2*).

*P. aeruginosa* is a gram-negative bacterium commonly found in natural and built wet environments (*3*) and is a well-established cause of sporadic piercing-related infections. Infections tend to occur (on average) 2–4 weeks after piercing procedures (*4*) and have historically been attributed to exposure of piercing sites to swimming pools and fresh water, lack of adequate preoperative/intraoperative antisepsis of piercing sites, poor hand hygiene, and using contaminated solutions during or after piercing procedures (*3*,*5*–*8*). However, limited reports exist on *P. aeruginosa* infection outbreaks related to piercing (*3*,*9*)

In late April 2021, the South Eastern Sydney Local Health District Public Health Unit in Sydney, New South Wales (NSW), Australia, was notified by an ear, nose, and throat clinician that 3 patients had sought treatment for *P. aeruginosa* infections at 2 local hospital emergency departments after ear piercings. All piercings took place on April 1, 2021, at a newly opened skin penetration facility located in southeastern Sydney. The identical pathogen, piercing date, and salon used by those 3 clients prompted an investigation into a potential common source. Environmental health officers initially inspected the implicated facility but did not find any store-specific practices or deficiencies that might have increased risk for piercing-related infections. We describe the methodology and outcome of this *P. aeruginosa* infection investigation, control measures implemented, and lessons learned.

## Methods

### Initial Case Definitions

We initially used broad case definitions to increase the sensitivity of active case finding. We defined a confirmed case-patient as a person who had a *P. aeruginosa* infection after a recent ear piercing and a probable case-patient as a person who had no cultures taken or no culture growth but had attended the implicated facility or other facilities of the same franchise or had used the same brand of aftercare solution.

### Emergency Department Syndromic Surveillance

The NSW Public Health Rapid Emergency, Disease, and Syndromic Surveillance (PHREDSS) system monitors treatment sought at most public hospital emergency departments in the state in near-real time (*10*). Patients are coded according to their illness and discharge destination (e.g., admitted or discharged home) (*10*). We identified possible infection cases at hospitals via PHREDSS by using keyword searches of triage text: (infect*|cellulitis*) and (earring*|earing*|pierc*|peirc*).

### Initial Investigations

We obtained details of procedures performed on confirmed case-patients, aftercare solutions used, notifications and complaints, and client lists from the initially implicated piercing salon. We identified additional cases of piercing-related infections in residents of greater Sydney, Wollongong, and Newcastle by examining the PHREDSS database.

### Statewide Investigations and Case Finding

Beginning on April 30, 2021, weekly PHREDSS line lists of emergency department visits and admissions for piercing-related infections were provided to other public health units (PHUs) across NSW. We asked PHUs to review the medical records of patients on the PHREDSS list who were within their local health district and to contact patients who had positive *P. aeruginosa* cultures from clinical swab samples; PHUs gathered demographic and hospitalization data, determined when and where the patients had their piercing performed, and what aftercare solution (brand and batch) they had used. We also asked PHUs to obtain any available bottles of aftercare solution(s) used by the patient for laboratory testing. We alerted other states about the outbreak in NSW and asked them to report any confirmed or probable cases.

### Microbiological Investigations and Whole-Genome Sequencing

Environmental health officers collected environmental samples and samples of aftercare solutions from the initial piercing salon for culture. We submitted specimens from case-patients and from opened and unopened bottles of aftercare solution to the Institute of Clinical Pathology and Medical Research–NSW Health Pathology for culturing and bacteria identification; positive culture isolates underwent whole-genome sequencing (WGS) by using the Illumina NextSeq platform (Illumina Corp., https://www.illumina.com), and their core genome multilocus sequence types (STs) were determined (*11*,*12*).

### Ethics

This study was conducted as a public health investigation under the NSW Public Health Act 2010. Therefore, ethics approval was not required.

## Results

The initial implicated facility was part of a nationwide franchise of piercing salons (franchise A), and this particular salon had opened on April 1, 2021, offering half-price piercings. The salon chain used Protat aftercare solution (Protat Tattoo Supplies, https://www.protatsupplies.com.au) from a single supplier. Unlike some aftercare solutions, Protat consists of natural preservatives, such as aloe vera, grapefruit seed extract, and *Melaleuca* *alternifolia* leaf oil, as well as saline containing benzalkonium chloride, which acts as an antimicrobial agent (*13*). The franchise’s usual practice was to apply Protat immediately after piercing from a bottle that was then offered to the client to take home (the same bottle was not used for >1 client).

### Descriptive Epidemiology

**Figure 1:**
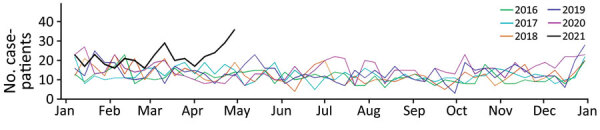
Weekly emergency department visits across New South Wales, Australia, by patients with piercing-site infections during 2016–2021 compiled for study of community outbreak of *Pseudomonas aeruginosa* infections associated with contaminated piercing aftercare solution. Data on emergency department visits and admissions for piercing-related infections were obtained from the New South Wales, Australia, Public Health Rapid Emergency, Disease, and Syndromic Surveillance system.

PHREDSS time series data showed an increase in emergency department visits for piercing infections in NSW beginning in April 2021 (Figure 1). Using the PHREDSS line lists, we identified 251 persons with a piercing-related infection via active case finding; 62 of those had *P. aeruginosa*–positive cultures.

We sent samples from 15 previously opened bottles of Protat aftercare solution retrieved from case-patients for microbiological testing. Samples from 10 Protat bottles with the implicated batch number were tested; 9 were positive for *P. aeruginosa*, and 1 had no bacterial growth. The implicated batch had a use by date of October 1, 2023, and had been supplied to tattooing and piercing establishments during February 16–May 11, 2021 (*14*). Samples from 5 additional Protat bottles with different batch numbers were also tested and had no bacterial growth (Table 1).

**Table 1:** *Pseudomonas aeruginosa* detection rates in opened and unopened product bottles in study of community outbreak of infections associated with contaminated piercing aftercare solution, Australia, 2021*

Source	No. bottles tested	No. (%) positive bottles
Opened bottles	15	9 (60)
Implicated batch	10	9 (90)
Other batch	5	0
Unopened bottles	11	3 (27)
Implicated batch	9	3 (33)
Other batch	2	0
Total	26	12 (46)

*No. (%) positive bottles indicates positive growth of *P.* *aeruginosa* after sampling and culture. Solution was manufactured by Protat Tattoo Supplies (https://www.protatsupplies.com.au).

We retrieved 11 unopened bottles of Protat aftercare solution from various chain stores in Sydney and Wollongong on May 14, 2021 (9 with the implicated batch number and 2 with different batch numbers). Samples from 3 unopened bottles of the implicated batch were positive for *P. aeruginosa* (850–2,000 CFU/mL); the remaining bottles had no growth (Table 1). We received additional samples from 48 unopened Protat aftercare bottles with the implicated batch number from South Australia; 10 of those bottles were cultured and had no bacterial growth.

**Figure 2:**
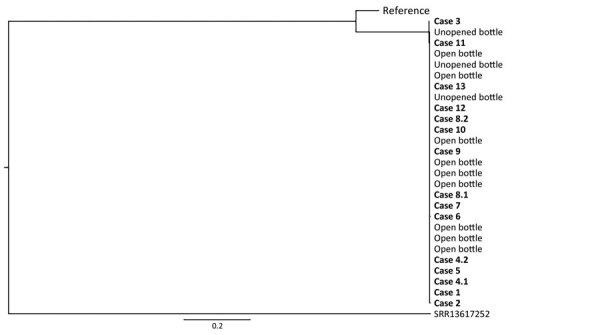
Phylogenetic analysis of *Pseudomonas aeruginosa* isolates collected in New South Wales in study of community outbreak of *P*. *aeruginosa* infections associated with contaminated piercing aftercare solution, Australia, 2021. Whole-genome sequencing was performed, and single-nucleotide polymorphisms were identified for 27 *P*. *aeruginosa* isolates from clinical specimens and opened or unopened bottles of Protat aftercare solution (Protat Tattoo Supplies, https://www.protatsupplies.com.au). Cluster analysis showed that all 27 sequences were genomically linked and belonged to sequence type 988. Reference indicates a representative sequence type 988 obtained from GenBank that was included in the analysis for comparison. The branch marked SRR13617252 indicates the *P*. *aeruginosa* mapping reference genome from the National Center for Biotechnology Information Sequence Read Archive database (https://www.ncbi.nlm.nih.gov/sra). Scale bar indicates nucleotide substitutions per site.

WGS was performed for 28 bacteria isolates (16 isolates from clinical samples, 3 from unopened bottles of Protat aftercare solution, and 9 from opened bottles of Protat aftercare solution); 27 of those isolates were *P. aeruginosa* and belonged to ST988. ST988 is a rare type that has not been identified previously in local isolate collections. Analysis identified 0–9 single-nucleotide polymorphism differences between the 27 isolate genomes (sequences with <25 single-nucleotide differences were regarded as a genomically linked cluster). Cluster analysis showed that all 27 submitted ST988 isolates were genomically linked. The remaining *P. aeruginosa* isolate was from a clinical sample and belonged to ST247 (Figure 2).

In total, we identified 13 case-patients who had *P. aeruginosa*–positive clinical isolates belonging to ST988 (2 case-patients had clinical samples taken from 2 different sites). Of the 13 case-patients with ST988 *P. aeruginosa* infections, 10 had used the implicated batch and 1 had used 2 different batches of Protat aftercare solution; information was not available for 2 cases. The case-patient with ST247 *P. aeruginosa* infection had used a different aftercare solution batch.

We reinterviewed the case-patient who reported using 2 different batches of Protat aftercare solution to confirm batch numbers. That case-patient had purchased those bottles 6 and 12 months earlier after other piercings and confirmed that additional aftercare solutions had not been purchased at the time of the latest piercing. However, a product was used in-store on their ear during the piercing procedure, and the case-patient was likely exposed to the implicated batch at that time.

### Outbreak Case Characteristics

After receiving the WGS analyses, we refined case definitions to be more specific. We used the confirmed case definition to determine associations with the suspected product, whereas the other case definitions tracked case incidence over time and assisted with ongoing case finding. We defined a confirmed case as a person in Australia with a body piercing infection caused by *P*. *aeruginosa* who had a piercing date after February 1, 2021, and ST988 detected in a clinical isolate. We defined a probable case as a person in Australia with a body piercing infection caused by *P*. *aeruginosa* who had a piercing date after February 1, 2021, but sequencing and typing data for a clinical isolate were not available; in addition, the piercing was performed at a franchise A store and the person had either used Protat aftercare solution or *P. aeruginosa* ST988 had been isolated from their aftercare product (regardless of store attended or product used). We defined a probable case either if a person in Australia had a body piercing infection caused by *P. aeruginosa *who had a piercing date after February 1, 2021, at a franchise A store but sequencing and typing data for a clinical isolate were not available; or the person had used Protat aftercare solution; or *P. aeruginosa *ST988 had been isolated from their aftercare product (regardless of store attended or product used). We defined a suspected case as a person in Australia with a body piercing infection caused by *P*. *aeruginosa* who had a piercing date after February 1, 2021, but the piercing location or aftercare product used was not known; a suspected case was also defined as a person in Australia with a body piercing infection and piercing date after February 1, 2021, but culture specimens were not collected or had no bacterial growth and the piercing site or use of aftercare solution was either not yet known, the store was not a franchise A store, or Protat aftercare solution was not used.

Of the 251 case-patients with piercing-related infections, 13 were confirmed, 40 were probable, 9 were possible, and 189 were suspected cases. Confirmed and probable cases predominantly comprised female patients (48/53, 91%) with a median age of 19.6 (range 15.5–59.6) years; 80% of those resided in metropolitan Sydney, Wollongong, or Newcastle. The median number of days between the piercing date and emergency department visit was 15 (range 3–35) days.

A higher percentage of confirmed and probable case-patients (30/53, 57%) required hospital admission compared with possible and suspected cases (9/198, 5%). In addition, confirmed and probable case-patients were more likely to have had piercings in cartilaginous areas, such as the tragus or helix, than possible and suspected cases and were much less likely to have been pierced in the earlobe or other noncartilaginous areas. We observed only minor differences in age, gender, and time (from piercing procedure to emergency department visit) between the confirmed/probable and possible/suspected groups. (Table 2)

**Table 2:** Differences in demographics, hospital admission status, and piercing characteristics according to case classification in study of community outbreak of *Pseudomonas aeruginosa* infections associated with contaminated piercing aftercare solution, Australia, 2021*

Characteristics	Confirmed or probable cases	Possible or suspected cases	Total no. (%) cases
Patient age, y, mean +SD	23.6 +10.2	21.9 +13.5	22.3 +12.8
Patient sex			
F	48 (90.6)	175 (88.4)	223 (88.8)
M	5 (9.4)	23 (11.6)	28 (11.2)
Piercing site			
Ear, tragus/antitragus	2 (3.8)	5 (2.5)	7 (2.8)
Ear, helix/antihelix	18 (34.0)	26 (13.1)	44 (17.5)
Ear, lobule	4 (7.6)	51 (25.8)	55 (21.9)
Ear, not specified	28 (52.8)	79 (39.9)	107 (42.6)
Other†	1 (1.9)	37 (18.7)	38 (15.1)
Hospital admission status			
Admitted	30 (56.6)	9 (4.6)	39 (15.5)
Discharged	23 (43.4)	189 (95.5)	212 (84.5)
No. days from piercing to hospital visit, mean +SD‡	14.1 +7.2	14.7 +9.8	14.4 +9.1

*Values are no. (%) except as indicated. †Includes breast, lip, nostril, and tongue. ‡Number of days could only be retrieved for a subset of case-patients through patient interviews and hospital records (n = 32 for confirmed or probable cases, n = 75 for possible or suspected cases).

All 13 confirmed case-patients attended either the initial implicated piercing facility or another franchise A facility and used Protat aftercare solution (Table 3). Of those, 10 case-patients were able to state specifically that they used the implicated batch of Protat aftercare solution; 1 stated that the Protat aftercare solution they had used came from a different batch, and 2 stated they had used Protat aftercare solution postpiercing but were unable to identify the batch number. The case-patient who claimed to have used a different batch of Protat aftercare solution was likely administered the implicated batch immediately after piercing in the store (Table 3). 

**Table 3:** Aftercare type and batch for confirmed and probable cases in study of community outbreak of *Pseudomonas aeruginosa* infections associated with contaminated piercing aftercare solution, Australia, 2021*

Source	Confirmed cases	Probable cases†	Confirmed and probable cases†
Protat	13 (100)	34 (85)	47 (89)
Implicated batch	10	20	30
Other batch	1	1	2
Unknown batch	2	13	15
Other brand	0	2 (5)	2 (4)
Not contactable	0	4 (10)	4 (8)
Total	13 (100)	40 (100)	53 (100)

*Values are no. (%). Implicated solution was manufactured by Protat Tattoo Supplies (https://www.protatsupplies.com.au). †Case definitions for probable cases include Protat aftercare use for franchise A piercings; this case definition was used to help monitor the burden of disease during this outbreak and monitor the involvement of batches other than the implicated batch.

### Outbreak Control Measures

On May 4, after identifying several additional *P. aeruginosa* piercing-related infections in clients who had attended franchise A stores across multiple NSW local health districts, the implicated franchisee agreed to cease using and selling Protat aftercare solution pending further investigation. NSW Health issued a clinician alert on May 13 to advise of an increase in hospital admissions because of *P. aeruginosa* infections after piercing procedures and to encourage clinicians to consider *P. aeruginosa* in patients with infections at piercing sites.

After confirmation of the presence of *P. aeruginosa* in unopened bottles of Protat aftercare solution, the distributor/manufacturer issued a voluntary recall of the contaminated batch from market shelves on May 14 and issued a product recall media release on May 31. On June 1, the Australian Competition and Consumer Commission published a recall notice for the implicated batch (*14*), which had been supplied to franchise A stores in NSW, Queensland, Victoria, and South Australia, as well as other tattooing and piercing establishments in all states of Australia and New Zealand. The commission also liaised with overseas regulators about the safety recall in Australia (*14*).

On May 28, after performing their own independent review, the manufacturer informed NSW Health that production/processing pathway testing of the implicated batch had detected a positive total microbial count, and results were PCR positive for *P. aeruginosa*. Root cause analysis conducted by the manufacturer identified several potential opportunities for contamination during manufacturing: handling of raw materials used for the manufacture of the product might have been compromised; flushing of the previous product out of the filling system might not have been performed sufficiently; the spray ball in the mixing tank might have been contaminated; the mixing tank’s lid might have been left open and unattended, potentially introducing contamination through water droplets; and the flow plate for tanks might have been contaminated when transfer connectors were switched between tanks. In addition, the prescribed microbial testing for this product was suspected to be insufficient.

**Figure 3:**
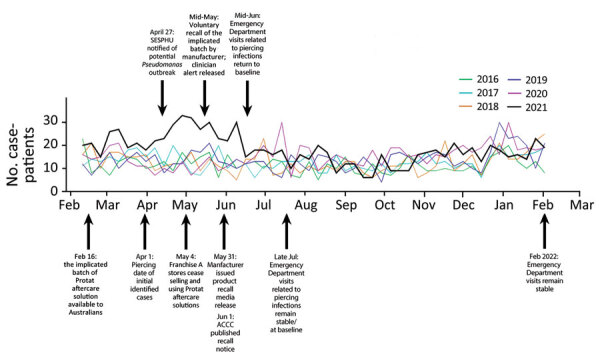
Timeline of key events during the community outbreak of *Pseudomonas aeruginosa* infections associated with contaminated piercing aftercare solution, Australia, 2021. Graph shows weekly numbers of *P*. *aeruginosa* infections in New South Wales during 2016–2021. Specific key events are shown for the 2021 outbreak of piercing-related *P*. *aeruginosa* infections. ACCC, Australian Competition and Consumer Commission; SESPHU, South Eastern Sydney Public Health Unit.

In response to the contamination, the manufacturer began preservative challenge testing on the product formula; increased microbial testing for opportunistic pathogens, including *P*. *aeruginosa*, *S*. *aureus*, and *Candida* spp.; and performed antimicrobial disinfectant fogging of the manufacturing facilities. They also planned to take additional preventive actions to address the risk for future contamination, such as reassessing cleaning and hygiene procedures and maintenance and operation of equipment and parts; providing refresher training for all manufacturing and production operators, emphasizing vigilance and aseptic techniques; and potentially reformulating the product on the basis of challenge test results. The effect of the outbreak control measures was evident in the decline in emergency department visits beginning in late May 2021 and their return to baseline levels by mid-June 2021 (Figure 3).

## Discussion

We found microbiological, environmental, and epidemiologic evidence linking a single batch of aftercare solution to a piercing-related *P. aeruginosa* infection outbreak across NSW during April–June 2021. The distinct whole-genome sequence type shared by 27 isolates from various sources, including clinical specimens and aftercare solution samples (both client-used and unopened bottles), established a single common source for this outbreak. Therefore, we successfully used WGS to establish a causative link between an aftercare product and a piercing-related *P. aeruginosa* infection outbreak.

Piercing-related *P. aeruginosa* infection outbreaks associated with aftercare solutions have been reported previously (*3*,*5*,*9*). In a 2016 outbreak in England, variable-number tandem-repeat (VNTR) typing was used to identify isolates from clinical samples collected from suspected case-patients and from environmental samples (opened and unopened bottles of aftercare solutions) to investigate possible links (*5*). The VNTR type for cases connected to that 2016 outbreak differed from cases unrelated to the outbreak and matched the VNTR type of isolates found in bottles of aftercare solution (*5*). In another outbreak in 2016 linked to a northwestern England piercing event, VNTR typing was also used to identify isolates from clinical samples of suspected case-patients, which matched isolates from water samples collected from the premises (*9*). Molecular subtyping was used to investigate an outbreak at an Oregon, USA, jewelry store in 2004 and successfully linked isolates from case-patients with *P. aeruginosa* piercing-related infections to isolates retrieved from a disinfectant bottle, as well as to isolates recovered from 2 workers and from wastewater located beneath sinks in the store (*3*).

Early recognition of the NSW outbreak and its subsequent effective management were partially attributed to the astuteness of the clinician who recognized and reported the cluster of *P. aeruginosa* piercing-related infections. Had this report not occurred, the outbreak might have gone unrecognized, and the cause might not have been identified as quickly, given that cases were dispersed geographically and the emergency department syndromic surveillance system was not routinely reviewed for this clinical syndrome. Furthermore, the successful investigation drew on the strengths of the NSW Public Health Network, which has conducted longstanding, real-time centralized surveillance of emergency departments, other health facilities, and public health units dispersed across the state. The network is well-placed to interact effectively with local patients and clinicians and also highlights effective coordination between clinical, public health, laboratory, and regulatory teams.

The first limitation of our study is that not all suspected case-patients had clinical isolates collected and not all probable cases had isolates retained for further characterization. Second, not all consumers had kept their bottles of aftercare solution or could recall the brand or batch number. Third, not all emergency departments in NSW are covered by PHREDDS; thus, some cases might have been missed. Nevertheless, sufficient evidence was available to issue timely clinician and consumer alerts and, eventually, a product recall, which prevented further infections.

Multiple outbreaks of *P*. *aeruginosa* infections from at least 2 continents have been caused by piercing aftercare products, suggesting that higher manufacturing standards might be required for such solutions. In Australia, although they are applied to recently penetrated skin, aftercare solutions are generally not regulated as therapeutic goods (*15*). Consumers expect aftercare solutions to be sterile, yet manufacturing processes reviewed in this investigation indicated that sterility could not be assured despite the manufacturer’s intentions. Management of this outbreak has shown the importance of quality control and sterility assurance in manufacturing such solutions. Existing measures routinely and effectively imposed on regulated therapeutic goods to reduce contamination risk should also be applied to aftercare solutions, such as objectionable organism and microbial risk assessments and sterility testing and control (*16*–*19*). Early detection of pathogen clusters linked to contaminated products and source attribution via genomic sequencing are pivotal in controlling outbreaks, as is effective communication with stakeholders, including clients, health professionals, piercing franchises, and manufacturers.
